# Molecular and structural perspectives on protein trafficking to the primary cilium membrane

**DOI:** 10.1042/BST20231403

**Published:** 2024-06-12

**Authors:** Vivek Reddy Palicharla, Saikat Mukhopadhyay

**Affiliations:** Department of Cell Biology, University of Texas Southwestern Medical Center, Dallas, TX 75390, U.S.A.

**Keywords:** cilia, ciliopathy, G-protein-coupled receptors, intraflagellar transport, trafficking, TULP3

## Abstract

The primary cilium is a dynamic subcellular compartment templated from the mother centriole or basal body. Cilia are solitary and tiny, but remarkably consequential in cellular pathways regulating proliferation, differentiation, and maintenance. Multiple transmembrane proteins such as G-protein-coupled receptors, channels, enzymes, and membrane-associated lipidated proteins are enriched in the ciliary membrane. The precise regulation of ciliary membrane content is essential for effective signal transduction and maintenance of tissue homeostasis. Surprisingly, a few conserved molecular factors, intraflagellar transport complex A and the tubby family adapter protein TULP3, mediate the transport of most membrane cargoes into cilia. Recent advances in cryogenic electron microscopy provide fundamental insights into these molecular players. Here, we review the molecular players mediating cargo delivery into the ciliary membrane through the lens of structural biology. These mechanistic insights into ciliary transport provide a framework for understanding of disease variants in ciliopathies, enable precise manipulation of cilia-mediated pathways, and provide a platform for the development of targeted therapeutics.

## Introduction

The primary cilium is a microtubule-based dynamic cellular appendage found in many cell types [1]. The microtubule axoneme core is templated from the mother centriole of the centrosome — the basal body. The ciliary membrane is contiguous with the plasma membrane. Primary cilia play crucial roles in signal transduction, serving as cellular antennae for sensing extracellular signals and mediating various signaling pathways in development and tissue homeostasis [2–6]. Defects in cilia or ciliary signaling result in syndromic diseases affecting multiple tissues called ciliopathies [7–9]. Excellent recent reviews on ciliary signaling in development and disease highlight the multifarious role of cilia in biology [10,11].

The ciliary membrane is distinct in protein and lipid composition from the rest of the plasma membrane. Multiple G protein-coupled receptors (GPCRs) [12,13], TRP-channel family proteins polycystin-1 and 2 [14,15], single-pass transmembrane protein fibrocystin [16], and multiple adenylyl cyclases [17–19] localize to cilia. Components of the hedgehog pathway, including the hedgehog receptor Patched, the pathway transducer Smoothened, and the GPCR GPR161, a repressor of the pathway, display dynamic localization in the ciliary membrane [20–22]. Furthermore, numerous lipidated membrane-associated proteins are enriched in the ciliary membrane. These include the palmitoylated atypical GTPase ARL13B [23,24], the farnesylated 5-phosphatase INPP5E [25,26], and the myristoylated proteins NPHP3 and Cystin [27,28]. The compartmentalization of these proteins in cilia is key to the transduction of cilia-specific signals.

Cilia-localized proteins are not exclusively present in the ciliary membrane. Rather, the small size of the cilium facilitates the enrichment of proteins in the compartment relative to the rest of the plasma membrane. The compartmentalization of factors in the small volume of the cilia enables effective reception and amplification of chemical or mechanical signals [29–31], generates transcriptional effectors [2,32], and modifies transcriptional outputs [33,34], fundamentally affecting downstream pathways driving morphogenesis [35–37]. Factors influencing the composition of proteins in the ciliary membrane include sorting of cargoes for cilia, transporting cargoes into cilia, cargo retrieval from cilia, retention of cargoes inside cilia, restriction of cargoes from reaching cilia, recycling in the endosomal compartment and loss of proteins through release of ciliary membrane vesicles [38–40]. Cargo retrieval from cilia is mediated by the BBSome [41–43], and has been discussed in recent reviews [38,44]. Here, we focus on molecular mechanisms involved primarily in the trafficking of proteins into the ciliary membrane and review recently discovered structural insights into the key players in this process.

## Ciliary gate

**Figure 1.**
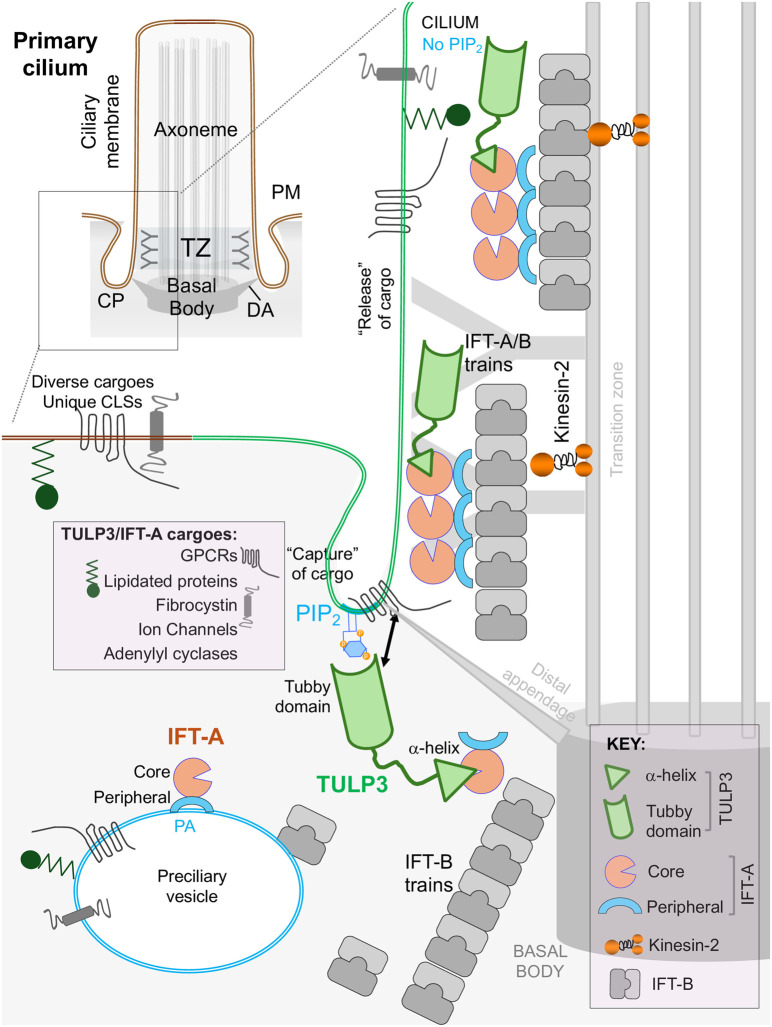
Key modules in trafficking to the ciliary membrane. **Left top**, parts of the primary cilium. The primary cilium transduces external stimuli into intracellular signaling directing tissue homeostasis. CP, Ciliary pocket; DA, Distal appendage or Transition fiber; TZ, Transition zone; PM, Plasma membrane. **Right**, steps in the trafficking of cargoes to cilia by TULP3 and IFT-A. PI(4,5)P_2_ bound TULP3 recruits diverse cargo ciliary localization sequences (CLSs) via its tubby domain, while the N-term α-helix captures IFT-A core subunits. IFT-A polymerizes on IFT-B trains. The IFT trains cross the TZ in a kinesin-dependent manner. Cargoes are released in the PI(4,5)P_2_ deficient cilia. Apart from strong localization in the base and tip of cilia, TULP3 localization is punctate inside cilia [49], suggesting that TULP3 might undergo IFT. Pre-ciliary vesicles with phosphatidic acid (PA) might bring in cargoes and IFT-A peripheral subunits [50,51] close to cilia prior to TULP3 capture in the PI(4,5)P_2_ ring between distal appendage/TZ [52].

The uniqueness of the ciliary membrane is generated and maintained by the presence of a barrier structure called the ciliary gate at the base of the cilium that regulates the entry and exit of proteins [45–48]. Two essential elements within the ciliary gate are transition fibers (distal appendages) and the transition zone (Figure 1). Transition fibers extend from the distal basal body to the base of the ciliary membrane, forming a structural bridge [53]. Distal to the transition fibers, the transition zone is composed of Y-links and the ciliary necklace [54,55]. Recent cryo-electron tomography (cryo-ET) structure of the flash frozen connecting cilia in vertebrate photoreceptors shows the circumferential span of the outer necklace beads with respect to the Y-links [55]. The overall architecture of the transition zone shows evolutionary conservation, despite species-specific differences [56], and is composed of MKS, NPHP, and CEP290 multi-protein complexes [57–59]. Functionally, the transition zone could serve as a membrane diffusion barrier that delays the passage of transmembrane proteins across the ciliary gate [60,61]. Proteins such as septins, localized to cilia and the transition zone, could further prevent outward diffusion of ciliary membrane components [60,61]. A condensed lipid zone at the ciliary base could also be restricting ciliary access [62]. The inversin compartment, another enigmatic periaxonemal subcompartment distal to the transition zone, is constituted by proteins that generate fibrillar structure [63]. Another periciliary subdomain, the ciliary pocket is composed of proteins that sustain high negative curvature [64,65] and is critical in ciliogenesis [66] and endocytosis [6].

Both the ciliary membrane and the ciliary base present specialized lipid composition [67,68]. The ciliary membrane lacks in PI(4,5)P_2_ levels, whereas PI(4,5)P_2_ and PI(3,4,5)P_3_ occupy distinct subdomains forming well-defined rings around the transition zone [52,69]. The spatial distribution of diverse ciliary lipid membrane subdomains may be influenced by the specific localization of ciliary lipid kinases and phosphatases. PI(4,5)P_2_ is depleted at the ciliary membrane because of ciliary enrichment of the 5-phosphatase, INPP5E [70,71], whereas local PI(4,5)P_2_ levels could be repleted by the presence of the phosphatidylinositol phosphate kinase PIPKIγ at the basal body [72,73].

## The role of IFT-A in protein trafficking to the ciliary membrane

The formation and maintenance of cilia, and the establishment of signaling pathways within cilia rely on a highly conserved trafficking mechanism known as intraflagellar transport (IFT) [1,30,74,75]. This intricate process facilitates the movement of proteins into, out of, and within cilia. The transport of proteins involves anterograde and retrograde IFT. Anterograde transport toward the ciliary tip is powered by kinesin-2 while retrograde transport is facilitated by dynein-2 [76,77]. Key to the functioning of IFT are the multisubunit complexes IFT-A and IFT-B. Soluble proteins including tubulin enter by diffusion into cilia [78,79] and can also be carried by IFT-B [80]. IFT-B is also responsible for the anterograde trafficking of dynein-2 and precursors of axonemal complexes [81,82]. In contrast, IFT-A is responsible for the retrograde movement of proteins from the ciliary tip to the basal body, inferred from the accumulation of IFT-B and other cargo in the ciliary tips in IFT-A mutants similar to dynein-2 mutants [83–85].

In addition to retrograde IFT, IFT-A has been implicated in the transport of transmembrane and membrane-associated proteins into cilia (Figure 1). These proteins include GPCRs, ion channels, other transmembrane proteins, and lipidated proteins [49,86–91]. IFT-A is composed of six proteins: IFT144 (WDR19), IFT140, IFT139 (TTC21B/THM1), IFT122, IFT121 (WDR35), and IFT43. Biochemical approaches showed subcomplexes in IFT-A: IFT144–IFT140–IFT122 form the core, while IFT139–IFT121–IFT43 form the peripheral subcomplexes [49,92]. The core subcomplex stays intact in the absence of the peripheral subunits [49,92], whereas the peripheral subunits also form a separate subcomplex [50]. Lack of core subunits prevents holo-complex assembly [49,92]. However, there are exceptions, as it has been shown that IFT140 is dispensable for IFT-A assembly [93] and that IFT43 also regulates the stability of the IFT-A holo-complex [93,94]. While the core subunits and the peripheral subunit IFT121 regulate ciliary membrane content, IFT139 does not affect transport to the ciliary membrane [49,86–91].

The initial evidence of IFT-A's role in ciliary cargo transport came from studies in *Drosophila* [86]. A TRPV ion channel, required to generate sensory potentials, was undetectable in *IFT140/RempA* and *IFT122/Oseg1* mutant chordotonal neuronal cilia, but showed accumulation of IFT-B proteins similar to *IFT dynein (btv)* mutants. Next, a study on IFT-A binding proteins using tandem immunopurification and mass spectrometry led to the identification of the tubby family protein TULP3 as an IFT-A core interacting protein in mammalian cells [49]. RNAi of IFT-A core subunits led to lack of TULP3 and GPCRs from cilia, whereas lack of any of the peripheral IFT-A subunits led to the accumulation of TULP3 in ciliary tips [49]. However, RNAi of core or peripheral IFT-A subunits resulted in the accumulation of IFT-B in ciliary tips [49]. Similarly, studies on IFT-A core mutants in mice described cargoes regulated by IFT-A [87,95,96]. The membrane proteins ARL13B, adenylyl cyclase III, and Smoothened failed to localize to primary cilia in the phenotypically strong IFT-A mutants–*Ift144^dmhd^* and *IFT144^twt^; IFT122^sopb^*. Trafficking of TULP3 was affected in the phenotypically weak *Ift122*^s*opb*^ mutant, although Smoothened trafficking to cilia remained unaffected [95]. Soluble proteins GLI2, SUFU, and KIF7 still localized to ciliary tips in these IFT-A mutants, while IFT-B components were accumulated in the ciliary tips [87].

A recent study on *IFT140* mutants in *Chlamydomonas* provides remarkable insights into IFT-A regulated ciliary membrane content. Lack of *IFT140* resulted in no flagella formation; however, expression of a truncated version of *IFT140* lacking the WD domains promoted short flagella formation with normal IFT-A and IFT-B localization in flagella [88]. Importantly, proteomic analysis of the membrane and matrix fractions from the *IFT140ΔWD* flagella showed altered flagellar localization of multiple classes of proteins: transmembrane proteins, myristoylated proteins, geranylgeranylated proteins, and GTPases [88]. In contrast, soluble protein cargoes were moderately increased as expected from defective retrograde IFT [88]. Another study used a powerful co-fractionation mass spectrometry technique, DIFFRAC, to identify the abundance of proteins upon *Ift122* loss in *Tetrahymena* [89]. The authors identified fibrocystin as a protein that decreased in abundance. To note, a fibrocystin cilia localization signal was previously shown to be regulated by TULP3 [97]. Further characterization of the differentially expressed proteins by performing *Ift122* loss in *Xenopus* motile multiciliated cells identified novel proteins, such as Pip5kl1 predicted to affect phospholipid composition, to be depleted from cilia [89].

Work from the Nakayama lab demonstrated distinct roles of IFT-A core vs peripheral subunit IFT139 [90]. *IFT139* knockout mammalian cells exhibited the accumulation of IFT-A, IFT-B at the bulging tips of cilia, similar to *IFT144* (core) knockout cells. However, ciliary localization of GPCRs, such as GPR161, SSTR3, MCHR1, and Smoothened remained unaffected unlike in *IFT144* knockout cells. While IFT139 is not required for ciliary entry of GPCRs, knockout of another IFT-A peripheral subunit, IFT121/WDR35, resulted in defective ciliary trafficking of multiple proteins including GPCRs and lipidated cargoes [91]. Interestingly, as in IFT140, the WD domains of IFT121 might play critical roles in cargo recognition as the TPR deleted version with only WD domains intact could still interact with the cargoes efficiently [91].

Several genetic mutations in IFT-A subunits have been documented in human diseases such as cranioectodermal dysplasia (CED), short-rib thoracic dysplasia (SRTD), nephronophthisis (NPHP), asphyxiating thoracic dystrophy (ATD), Senior–Loken syndromes (SLSN), retinitis pigmentosa (RP), and the autosomal dominant polycystic kidney disease (ADPKD) spectrum [98–103]. Some of the disease phenotypes in IFT-A mutants could be because of defects in cargo trafficking. For example, some of the point mutations and truncation mutations in *WDR35* that do not affect IFT-A complex formation, have been shown to cause defects in cargo interactions [91] and the disease phenotypes might result from the lack of proper cargo trafficking.

It is important to emphasize that the accumulation of membrane proteins in existing cilia triggered by signaling pathways can be regulated by IFT-independent mechanisms. For example, the entry of the ciliary adhesion protein SAG1 in *Chlamydomonas* is independent of anterograde IFT [104]. Similarly, the dynamics of GPR161 and Smoothened levels in cilia during hedgehog signaling is determined by a balance between the rates of entry and exit of these GPCRs [105,106].

## TULP3 — a central adapter in IFT-A mediated ciliary protein trafficking

The tubby family protein TULP3 was discovered as an interacting protein of the human IFT-A complex using tandem affinity purification-mass spectrometry [49]. TULP3 is now established as a central adapter involved in IFT-A mediated ciliary cargo trafficking [49,97,107]. TULP3 has an N-terminal helix that binds to IFT-A and a C-terminal tubby domain that binds to PI(4,5)P_2_ and membrane cargoes thereby acting as a bridge between IFT-A and ciliary cargoes (Figure 1). TULP3 is required for the ciliary trafficking of many cargoes that belong to a wide variety of proteins. These include multi-pass transmembrane cargoes such as class A GPCRs (such as GPR161, MCHR1, SSTR3, etc.) and polycystins, single-pass transmembrane protein Fibrocystin, and multiple membrane-associated lipidated proteins including ARL13B, and ARL13B-dependent lipidated cargoes such as INPP5E, NPHP3, and CYS1 [49,97,107–110]. In some scenarios such as the neural tube and brain neurons, TULP3 functions redundantly with the founding tubby family member Tubby (TUB) in the trafficking of cargoes [97,111,112]. The *Drosophila melanogaster* homolog of TUB, dTULP, regulates the localization of TRP channels in auditory neuronal cilia [113]. In *Caenorhabditis elegans*, the TUB homolog TUB-1 determines the localization of GPCRs and cyclic nucleotide-gated channels to olfactory neuron cilia [114].

In line with a role of TULP3 in trafficking multiple cargoes to cilia, TULP3 has been implicated in neural tube patterning [21,115], renal and liver cystogenesis [109,116,117], adipogenesis [118,119], and spina bifida [120–122]. Kidney-specific *Tulp3* knockouts result in embryonic kidney cysts and these cysts are intermediate between those caused by loss of cilia or polycystins [109,110,123]. Ciliary trafficking of multiple lipidated proteins including ARL13B, INPP5E, NPHP3, and CYS1 was defective in *Tulp3* knockout cells and conditional knockout kidneys [107,109,110]. Noteworthy among these proteins is ARL13B, a protein associated with Joubert syndrome, known to induce cystic renal disease upon conditional deletion [124,125] or lack of trafficking to cilia in mouse renal epithelial cells [126]. Deletion of *Inpp5e* [127], *Nphp3* [128], or *Cys1* [129] in renal epithelial cells similarly leads to cystic phenotypes. Interestingly, these proteins are lost from *Tulp3* conditional knockout kidney cilia with varying kinetics [107]. To note, *Tulp3* conditional knockout does not result in gross ciliary defects unlike IFT-A knockouts [107,109,110]. Thus, modulating TULP3 is an effective way to study protein trafficking to cilia while keeping the cilia intact.

Short sequences in multiple TULP3 cargoes have been identified to act as ciliary localization or targeting sequences (CLSs). These are both necessary and sufficient for ciliary targeting in a TULP3-dependent manner [49,70,97,107]. Although the CLSs recognized by TULP3 are diverse, an examination of multiple TULP3 cargoes unveils two potentially shared structural characteristics among some of these diverse CLSs. Firstly, many of the transmembrane protein cargoes have sequences with secondary helical structures that interact with TULP3. Secondly, these sequences need to be near the membrane, enhancing their interactions with the tubby domain. For example, the CLS (VKARK) in GPR161 IC3 forms an extended TM5 helix as shown in a recently reported high-resolution structure [71]. Likewise, the CLS sequence (KTRKIKP) of fibrocystin could be part of an extended TM domain [130]. Similarly, an N-terminal amphipathic helix in ARL13B that promotes its membrane association acts as a TULP3-dependent CLS [107]. A recent study shows that trafficking of the mu opioid receptor, a GPCR, to cilia is TULP3-dependent but requires a 17-residue C-terminal sequence also involved in recycling [131]. To note, the RVxP motif has been identified as a CLS in some ciliary localized proteins including ARL13B [132] and PC1 and PC2 [133,134]. However, the RVxP motif was initially identified to be involved in the post-Golgi trafficking of Rhodopsin [135,136]. Additionally, although the RVxP (RVEP) motif is required for the ciliary localization of ARL13B [132], this sequence is not required for the interaction of ARL13B with TULP3 [107]. Therefore, RVxP motifs in cilia-destined proteins might function at upstream steps during the intracellular trafficking of these proteins [137], much before reaching the TULP3 interaction step.

**Figure 2.**
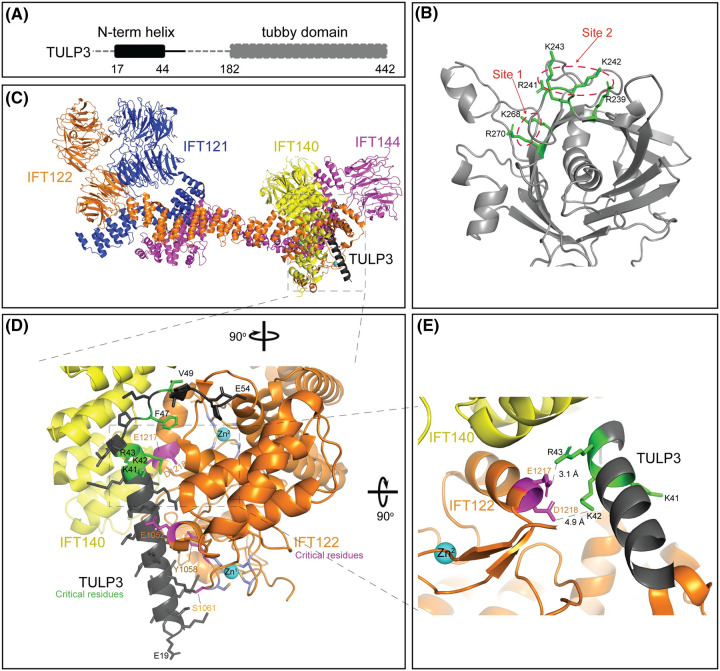
Structural basis of TULP3 interactions with phosphoinositides and IFT-A. (**A**) Cartoon showing the IFT-A interacting N-terminal region and the PI(4,5)P_2_/cargo-interacting tubby domain of TULP3 (Uniprot O75386). (**B**) Residues at the PI(4,5)P_2_ binding regions (green) of the tubby domain of TULP3 (gray) are shown. Site 1 and Site 2 are marked with red dashed lines. Site 1 was initially described in the crystal structure of the tubby domain of TUB bound with a PI(4,5)P_2_ analog [138], and site 2 was later inferred from molecular dynamics simulation studies [139]*.* The tubby domain of TULP3 is based on AlphaFold. (**C**) Cryo-EM structure of TULP3–IFT-A complex (without IFT139) (PDB 8FH3) [93]. The alpha-helix and loop at the N-terminus of TULP3 are shown in black. (**D**) TULP3 interaction interface with IFT-A shows the alpha-helix and loop of TULP3 with the critical amino acids highlighted in TULP3 (K41, K42, R43, F47, V49 in green) [93] and IFT122 (E1052, Y1058, S1061, E1217, and D1218 in magenta) [140]. Note the two Zn fingers (with two Zn molecules numbered as Zn^1^ and Zn^2^) in IFT122 that are important in the proper folding of the TULP3 interaction region. Cysteines in the Zn fingers are marked in blue with sulfhydryl groups shown in yellow. (**E**) Atomic interactions between K42 and R43 of TULP3 and E1217 and D1218 of IFT122 with distance measurements shown in dashed lines. Note the adjacent Zn finger 2 of IFT122.

The structural details of the tubby domain-PI(4,5)P_2_ interactions are well established [138,139] (Figure 2A,B), and the TULP3-PI(4,5)P_2_ interaction is a critical step during ciliary protein trafficking [49,97,107]. The occurrence of a PI(4,5)P_2_ between the transition zone and distal appendages [52,67–69,72,73] suggests that this might be the location where TULP3 engages in cargo recognition by TULP3 (Figure 1). Lack of PI(4,5)P_2_ in the ciliary membrane suggests that TULP3 is released from the membrane inside the cilium which in turn releases the membrane cargoes and likely prevents reengagement of the membrane cargoes inside the cilium (Figure 1). In agreement with this, INPP5E inactivation causes ciliary accumulation of TULP3 and IFT-A and TULP3-dependent cargoes such as GPR161 [141,142]. Furthermore, mutations in INPP5E, especially those removing the CAAX motif crucial for ciliary membrane localization without affecting 5-phosphatase activity, are associated with ciliopathies [25,143], underscoring the significance of INPP5E's ciliary localization.

How TULP3 recognizes these diverse cargoes is not well understood. The C-terminal tubby domain of TULP3 is involved in interaction with cargoes and PI(4,5)P_2_ [49,97,107]. Multiple mutations in the *TULP3* tubby domain have been identified in mice and humans with fibrocystic hepatorenal disease. The *Tulp3* tubby domain *K407I* mutation in mice has been shown to cause kidney cystogenesis and disrupt ciliary trafficking of ARL13B without affecting TULP3 protein levels [110]. Two homozygous *TULP3* mutations, *R382W* [117] and *R408H* [116], presenting with fibrocystic renal and hepatic disease, disrupt protein trafficking to the ciliary membrane. The identified *TULP3* patient mutations are distinct from the PI(4,5)P_2_ binding pocket [138,139], and understanding the molecular and structural intricacies of TULP3-cargo interactions emerges as an intriguing avenue for further investigation.

## Structural insights into IFT-A complexes and conformations

Recent structural studies on IFT-A complexes using cryogenic electron microscopy (cryo-EM) provided molecular details of IFT-A assembly, IFT-A train formation, and cargo transport at the atomic level. Multiple groups reported high-resolution structures of IFT-A complexes. Alan Brown's lab provided structures of the IFT-A complex from *Leishmania* [51]. Ming Lei and colleagues provided structures of the IFT-A complex from *Tetrahymena* [144]. Anthony Roberts and colleagues [140] provided structures of reconstituted human IFT-A complex modules. Ji Sun's lab provided structures of the IFT-A complex and TULP3 by reconstitution of human proteins [93].

**Figure 3.**
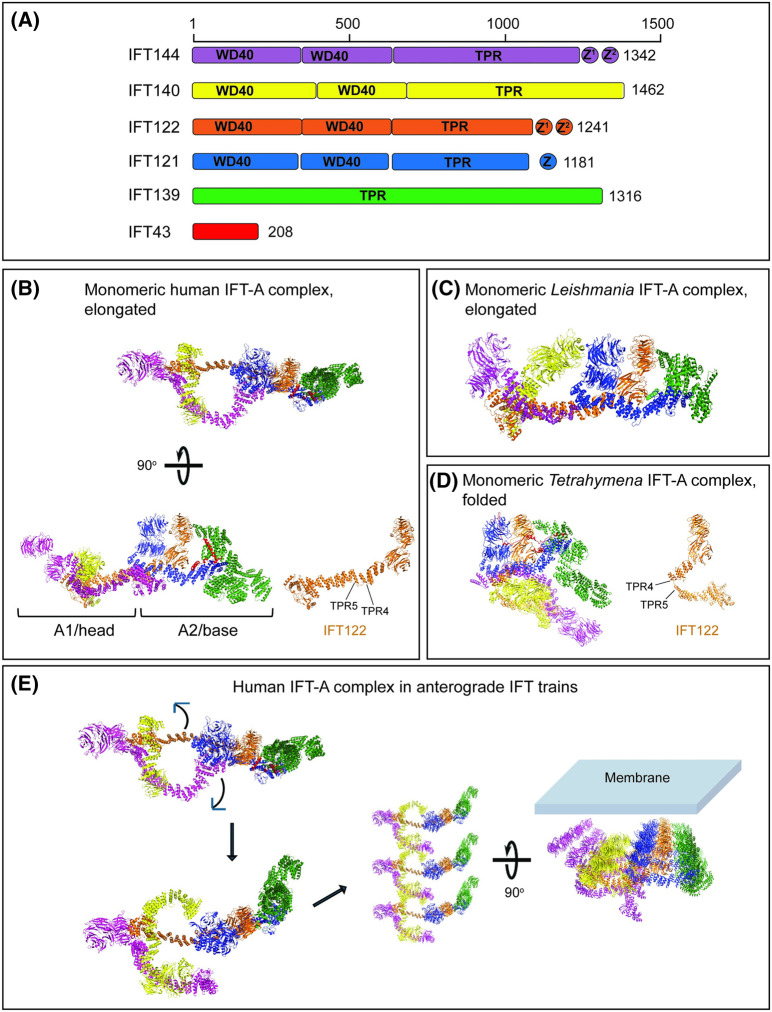
IFT-A cryo-EM structures and different known conformations. (**A**) Subunits of the human IFT-A complex showing WD, TPR domains, and Zinc fingers. (**B**) Monomeric human IFT-A complex (PDB 8FGW) shows lariat-like elongated conformation [93]. (**C**) Monomeric IFT-A complex conformation in *Leishmania* (PDB 8F5P) showing elongated conformation [51]. (**D**) Monomeric IFT-A complex conformation in *Tetrahymena* (PDB 8HMD and 8HMF) that shows a folded conformation from bending in the TPR repeats 4 and 5 of IFT122 [144]. IFT122 (orange) is shown isolated in the structural orientations to show conformational changes in (**B**) and (**D**). (**E**) Conformational changes predicted in IFT-A complex (PDB 8BBG) during IFT-A train formation [51,93,140,144]. Docking of the high-resolution human IFT-A structure on to the cryo-ET maps of *Chlamydomonas* IFT-A trains [145] help orient the IFT-A complex with respect to the ciliary membrane. This orientation shows that the WD domains of IFT-A face toward the membrane.

Structures of IFT-A from all organisms showed similar features with some differences [51,93,140,144]. All the IFT-A proteins except IFT43 are composed of WD domains and/or TPR repeats (Figure 3A–D). The TPR repeats of the IFT-A proteins face one side of the IFT-A complex while the WD domains face the other side. IFT-A is composed of two rigid modules with similar sizes, which have been designated as the head (IFT-A1) and the base (IFT-A2) modules of IFT-A [51,140,144]. The head module consists of IFT140, IFT144, and the C-terminal half of IFT122, whereas the base module consists of IFT121, the N-terminus of IFT122, IFT139, and IFT43. The elongated IFT-A complex adopts a lariat-like structure with IFT-122 acting as a spine (Figure 3B). IFT43 acts as a clamp holding the complex together. Zinc binding domains (ZBDs) are noticed in many of the IFT-A subunits that likely maintain conformational stability [51,93,144].

The WD40 domains within IFT140 prove challenging for *de novo* model construction due to their excessive flexibility and limited interaction with the remaining IFT-A complex components. This observation offers a potential explanation for the partial restoration of flagella formation and flagellar localization of IFT-A in *Chlamydomonas* by truncated IFT140 lacking WD40 domains [88]. Similarly, interaction studies showed that the TPR deleted version of IFT121 does not interact with IFT-A but could still interact with the cargoes, suggesting that the WD domains of IFT121 interact with the cargoes even in the absence of an intact IFT-A complex [91]. The TPR-like domains found in IFT122 and IFT144 play a crucial role by structurally connecting the two opposite sides of the lariat loop within the IFT-A complex [93], linking the core and peripheral subcomplexes [49,92]. Comprised of TPR or TPR-like repeats, IFT139 adopts a right-handed superhelix configuration and is positioned distally within the IFT-A complex. This structural organization corresponds with the finding that IFT139 is not essential for the assembly of IFT-A [90,93]. However, IFT139 is critically important in the formation of trains.

The IFT-A head module (IFT-A1) has relative flexibility with respect to the base module (IFT-A2) [140]. A stark variation in IFT-A complex structures is a folded conformation identified in the *Tetrahymena* (Figure 3D) in addition to the elongated conformation, in accordance with the fast and slow-migrating states captured by the native-PAGE analysis [144]. The individual head and base modules are almost identical in the folded state with respect to the elongated state. The bending happens in between the 4th and 5th TPR repeats of IFT122 resulting in the head module to fold back onto the base module [144]. This conformation allows proximity between the WD and TPR domains of IFT140 (Figure 3D). The biological significance of this folded conformation is not currently understood but could be involved in the assembly of retrograde IFT trains or cargo capture.

Another interesting aspect of IFT-A is the formation of trains. Gaia Pigino's laboratory achieved the initial resolution of cryo-ET structures pertaining to anterograde IFT particles in *Chlamydomonas* [146]. Recently, they further enhanced the detailed structures of IFT-A to 20.5 Å [145]. By analyzing cryo-ET maps of IFT-A trains and high-resolution cryo-EM structures of IFT-A monomers, it becomes evident that the fitting of the monomeric IFT-A complex into IFT-A trains involves multiple conformational changes [51,93,140,144] (Figure 3E). This process includes the transformation of the lariat structure of IFT-A, while preserving the invariant IFT122 spine, into a trident shape, which establishes close interactions between the WD motifs of IFT140 and WDR35 from neighboring subunits. Docking of the high-resolution human IFT-A structure on to the cryo-ET maps of *Chlamydomonas* IFT-A trains also helps orient the IFT-A complex with respect to the membrane (Figure 3E). This orientation shows that the WD domains of IFT-A face toward the membrane while the TPR regions are on the opposite face [51,93,140,144] (Figure 3E).

Furthermore, comprehensive cryo-ET studies in *Chlamydomonas* [147] and single molecule imaging in cilia of *C. elegans* chemosensory cilia [148] offer remarkable insights into the *in situ* assembly of oligomeric trains during anterograde IFT. In contrast, the structural basis of retrograde IFT trains is not currently known. The anterograde IFT trains are assembled in a stepwise fashion as they are anchored in the transition zone and ciliary base [147]. Dynein 2 is carried in an inactive conformation in the anterograde trains [146]. Kinesin-2 motors only associate with the anterograde IFT trains in a late stage of their assembly [148]. IFT-A oligomerization takes place on pre-existing IFT-B trains, suggesting that IFT-B trains might initiate IFT-A train formation [145,147]. Interactions between IFT-B and IFT-A are likely responsible for inducing conformational changes in IFT-A, facilitating its oligomerization. In agreement with the structural data, the N-terminus of the IFT-B subunit IFT74 is required to recruit IFT-A to IFT-B for assembly of IFT trains at the base of the *Chlamydomonas* flagella [149]. Furthermore, IFT-A trains are absent from *Ift139* knockouts, despite having intact assembly of IFT-B trains [146]. Mutants in *Ift139* that do not assemble IFT-A trains, still show steady-state levels of cargoes in cilia similar to wild-type [90]. Therefore, trafficking of TULP3–IFT-A cargoes might not require anterograde IFT-A train formation.

*In vitro* studies demonstrate that purified *Chlamydomonas* IFT-A peripheral subcomplex (IFT139/121/43) and the *Leishmania* IFT-A holo-complex can directly bind to specific lipids, including phosphatidic acid (PA) [50,51]. Furthermore, the IFT-A peripheral subcomplex (IFT139/121/43) binds to PA-containing liposomes [50]. Structural studies suggest that the TPR regions of the IFT-A core components and the TPR regions of IFT139 face and likely interact with the liposome membrane [51]. The context of these TPR interactions with respect to pre-ciliary vesicles [150–152] and whether the IFT-A subunits link with cargoes in pre-ciliary vesicles is an intriguing area for future investigation (Figure 1).

## Structural basis of TULP3–IFT-A interactions

Initial studies on TULP3 binding to IFT-A inferred that the N-terminus alpha-helical region binds to the IFT-A core [49]. The N-terminus alpha-helical region in TULP3 is highly conserved and present in some of the tubby family members, Tubby and TULP2, but not present in TULP1 and TULP4. This alpha-helical region was shown to involve binding of Tubby and TULP2 to IFT-A using TAP-MS and was further confirmed in the case of Tubby using *in vitro* binding to IFT-A [49,97].

A recent cryo-EM study solved the structure of human IFT-A in complex with human TULP3 N-terminus (aa19–54) [93]. The cryo-EM structure shows that the TPR domains of IFT140 and TPR/zinc finger domains of IFT122 interact with an alpha-helix and an adjacent loop region in the TULP3 N-terminus (Figure 2C,D). The electrostatic surface representation showed that the helix binding surface in IFT-A is negatively charged, and the loop-binding interface has mixed chemical properties with both hydrophilic and hydrophobic residues. Multiple residues in the helix and loop regions of TULP3 N-terminus interface with IFT-A (Figure 2D). Mutational analysis showed that residues in both the helix and loop regions are critical in mediating IFT-A interactions and ciliary trafficking of both GPR161 and ARL13B [93]. Corresponding residues in IFT122 that were inferred from AlphaFold modeling and *in vitro* binding, especially D1218 and E1217 [140], have close interactions with critical TULP3 residues at the C-end of the alpha-helix, K42 and R43, respectively [93] (Figure 2D,E). Importantly, the Zn fingers in IFT122 are adjacent to the TULP3 binding region facilitating proper interactions (Figure 2D,E). Thus, TULP3 N-terminus-IFT-A structure provides molecular details of TULP3–IFT-A interactions and their role in ciliary protein trafficking [93]. The interactions between the cargo-interacting tubby domain, IFT-A, and cargoes are important questions that currently remain unanswered.

## Conclusions and future directions

Research from numerous laboratories in the past two decades was pivotal in providing a parts list of ciliary membrane components and identify key players in ciliary compartmentalization. Recent advances in cryo-EM and structural biology provide important insights into ciliary transport processes. These mechanistic insights provide a framework for understanding disease variants in ciliopathies and allow the design of tools to precisely manipulate cilia-mediated pathways. We propose that addressing the following questions will be key to new advances in the field. First, what mechanisms regulate protein movement across the transition zone? Second, is the retrograde trafficking function of IFT-A separable from the cargo trafficking function? For example, is the train formation function of IFT-A required both for cargo transport and retrograde IFT? Third, is the role of IFT-A in cargo trafficking only from direct binding to the TULP3 N-term α-helix or do TULP3 tubby domain and IFT-A provide additional interfaces in cargo transport? Fourth, does TULP3 undergo IFT, and how is TULP3 prevented from reengaging cargoes in cilia? Fifth, new findings on the movement of IFT-A to the ciliary base show that IFT-A is often not near the membrane in assembling IFT trains [147,153]. How are these dynamics likely to impact the timing of TULP3-cargo binding? Finally, what are the mechanisms and locations of interactions between cargo-loaded pre-ciliary vesicles and the IFT-A complex? Understanding the molecular and structural features of ciliary transport will not only allow precise manipulation of cilia-specific signaling but also has immense potential for the design of cilia-targeted therapeutics.

## Perspectives

- The primary cilium, long considered vestigial, is now established as a paradigmatic subcellular compartment at the nexus of multiple cellular pathways and diverse morphogenetic trajectories.
- Recent technological advances in cryo-EM and structural biology are providing remarkable insights into the ciliary trafficking modules. These mechanistic insights into ciliary transport provide a framework for understanding of disease variants and their roles in ciliopathies.
- Elucidation of the structural and spatiotemporal dynamics of the trafficking complexes with ciliary cargoes has immense potential for the development of therapeutics targeting cilia-specific signaling in the near future.

## Open Access

Open access for this article was enabled by the participation of University of Texas Southwestern Medical Center in an all-inclusive *Read & Publish* agreement with Portland Press and the Biochemical Society under a transformative agreement.

## Abbreviations

cryo-EM: cryogenic electron microscopy
cryo-ET: cryo-electron tomography
GPCR: G-protein-coupled receptor
IFT: intraflagellar transport
PI(4,5)P_2_: phosphatidylinositol 4 5-bisphosphate
TULP3: tubby-like protein 3
ZBD: zinc binding domain
